# Clinical measures of paediatric foot posture: a critical review

**DOI:** 10.1186/1757-1146-4-S1-P20

**Published:** 2011-05-20

**Authors:** Angela Evans, Sheila Scutter, Katherine Kneebone

**Affiliations:** 1 School of Health Science, University of South Australia, North Terrace, Adelaide, 5000, Australia

## Background

The paediatric flat foot is a common concern and has long been regarded as a problem, and feared to be potentially disabling. Definition of what exactly constitutes a flat foot remains surprisingly debatable, given its common presentation. Estimates of flat foot prevalence in children have been influenced by the varied the methods of assessment used to assess the foot posture, and the subsequent criteria employed to delineate feet as flat versus not flat. The aim of this literature review was therefore to evaluate the techniques of foot posture assessment in children and the reported reliability and validity of these measures.

## Method

A systematic search of electronic databases including Medline (1966-present), CINAHL, SportDiscus, Embase, Scopus and Pedro occurred between 02/01/10 and 14/08/10. Eligible articles were selected according to pre-determined criteria. Methodological quality was evaluated by use of the Quality Index as described by Downs & Black, followed by critical analysis according to outcome variables.

## Results

The most widely reported measures of paediatric foot posture were footprints and measures of the heel angle and arch height. The current evidence suggests that the reliability of all measures of paediatric foot posture is highly variable and mostly poor to moderate. The only measures on which validity has been explored are navicular height and footprints.

## Conclusion

Whilst no definitive conclusions can currently be drawn from the existing evidence, the trend from the current literature indicates that static paediatric foot posture may be best-assessed using RCSP, NH or FPI-6. However the relationship between static measures and pain, static measures and gait function remain largely unsubstantiated in children. The direction of future research is to establish a universal method of assessment of paediatric foot posture, and the subsequent relevance of foot posture to pain and function across age groups. Continuation of research in this field will enable targeting of design parameters towards variables that are supported by evidence, and which may directly advance clinical decision-making.

